# Prehabilitation Practices for Paediatric Haematopoietic Stem Cell Transplantation: A Survey and Patient and Public Involvement Study of Healthcare Professionals

**DOI:** 10.3390/nu18172762

**Published:** 2026-08-24

**Authors:** Hala AbuSalameh, Raquel Revuelta Iniesta, Deborah Rowley

**Affiliations:** 1 Public Health and Sports Sciences, Faculty of Life and Health Sciences, Medical School, University of Exeter, Exeter EX1 2LU, UK; ha524@exeter.ac.uk (H.A.); deborah.rowley2@nhs.net (D.R.); 2 Haematology and Oncology, Sheffield Children’s NHS Foundation Trust, Clarkson Street, Broomhall, Sheffield S10 2TH, UK

**Keywords:** prehabilitation, haematopoietic stem cell transplantation, children and young people, healthcare professionals, patient and public involvement

## Abstract

**Background/Objectives**: Haematopoietic stem cell transplantation (HSCT) causes substantial morbidity in children and young people (CYP). Although prehabilitation benefits adults with cancer, its role in paediatric HSCT remains underexplored. This study aimed to describe current pre-transplant assessment and supportive care practices across paediatric HSCT centres, explore healthcare professional perspectives on prehabilitation implementation, and integrate patient and public involvement (PPI) to inform future intervention design. **Methods**: A cross-sectional survey was administered to healthcare professionals across 16 paediatric HSCT centres in the UK and the Republic of Ireland, alongside semi-structured PPI discussions conducted with eight children, young people, and caregivers with direct experience of paediatric HSCT. Survey data were analysed descriptively, free-text responses by qualitative content analysis, and PPI by reflexive thematic analysis. **Results**: Forty-nine eligible responses were received from healthcare professionals. Formal prehabilitation services were reported by 27 (55.1%) respondents, with substantial within-centre variation. Nutritional advice was the most consistently delivered component, 36 (72%), whilst physical activity interventions were the least consistently provided, 13 (26%). Workforce limitations were identified as the dominant barrier by 37 (95%) respondents. PPI findings described provision as reactive and inconsistent, with families expressing preference for flexible, hybrid, and family-centred delivery models. **Conclusions**: Prehabilitation provision in paediatric HSCT is variable, often informal, and limited by workforce capacity. CYP and caregivers expressed a desire for prehabilitation, particularly through flexible, hybrid (face-to-face and online), and family-centred approaches. Future research should prioritise co-design and feasibility testing of safe, individually tailored programmes that can be integrated into paediatric HSCT pathways.

## 1. Introduction

Haematopoietic stem cell transplantation (HSCT) is an intensive, potentially curative treatment for children and young people (CYP) with high-risk or relapsed haematological malignancies and selected non-malignant conditions, including inherited metabolic disorders, bone marrow failure syndromes and primary immunodeficiencies [1]. In paediatric oncology, HSCT is primarily used for patients at high risk of relapse or without sustained remission following first-line treatment [2]. Advances in conditioning regimens, donor selection, human leukocyte antigen matching and supportive care have improved survival, with one large multicentre study reporting an increase from 89% to 96% over ten years [3]. Nevertheless, HSCT carries substantial acute risks, including graft-versus-host disease, infection and organ toxicities, alongside long-term endocrine, neurosensory and musculoskeletal morbidity. Among childhood HSCT survivors, 79% report at least one chronic health condition and 25% a severe or life-threatening condition [4,5]. Associated impairments in physical function, nutrition and psychological wellbeing may persist into survivorship and reduce health-related quality of life [6].

Many CYP referred for HSCT have substantial cumulative treatment exposure from repeated chemotherapy and prolonged illness, resulting in physical, nutritional and psychological vulnerabilities [7]. Pre-transplant, they demonstrate impaired fitness, muscle strength (50% lower than healthy peers), mass and function, increased fatigue, and poorer quality of life [8,9]. At admission, leg-extension and handgrip strength are approximately 52% and 48% below reference values, respectively [10]; 11% meet criteria for risk of sarcopenia and up to 37.5% have reduced muscle mass [11]. Nutritional deficits are also common, driven by prior chemotherapy, reduced oral intake, systemic inflammation and treatment-related catabolism [12]. Psychological morbidity includes anxiety disorders in nearly 30% before and after HSCT, while mood disorders increase from 10% at baseline to 14% at 12 months [13]. These vulnerabilities may impair treatment tolerance, increase complications, prolong hospitalisation and delay recovery [14,15]. The pre-transplant period therefore offers an underused opportunity to optimise readiness and mitigate treatment-related decline [6].

Prehabilitation is a proactive, multimodal strategy that enhances physical fitness, nutritional status and psychology wellbeing before treatments [16,17]. Multidisciplinary programmes typically integrate exercise, nutritional optimisation and psychological support tailored to individual and clinical needs [18]. In adult oncology, prehabilitation is associated with improved functional capacity and quality of life and may reduce complications and hospital stay [16]. Evidence in paediatric HSCT, however, is limited to few, predominantly retrospective and heterogeneous studies, with no multimodal interventions identified [6]. No standardised model exists for CYP, and routine provision across these domains remains poorly understood. This is an important evidence gap given their distinct developmental and physiological needs.

Before structured prehabilitation interventions are developed and evaluated, current clinical practice must be understood. Mapping pre-transplant assessment and supportive care across centres can identify variation, strengths and development needs, informing interventions feasible for routine care. Meaningful involvement of CYP and families is equally essential. Patient and public involvement (PPI) can strengthen study design, recruitment and response rates, and facilitate clinical implementation [19]. Within paediatric HSCT specifically, where the demands of treatment extend well beyond the individual child to the wider family unit, this multi-stakeholder approach spanning healthcare professionals, CYP, and families represents the most robust foundation for driving meaningful and sustainable service change [6].

To date, no study has systematically examined prehabilitation-related practices in paediatric HSCT across UK transplant centres. Addressing this gap is essential to inform the development of evidence-based, patient-centred, and implementable prehabilitation strategies. Therefore, this study aimed to describe current pre-transplant assessment and supportive care practices across paediatric HSCT centres including nutritional, physical activity, and psychological support. In addition, the study explored healthcare professionals’ perspectives on existing gaps in care, the feasibility of implementing prehabilitation within routine pathways, and key components that should be included in future interventions, informed by PPI.

## 2. Materials and Methods

### 2.1. Study Design

This multicentre cross-sectional survey was designed to explore current prehabilitation practices and the perspectives of healthcare professionals working within UK and the Republic of Ireland paediatric HSCT centres, alongside perspectives of CYP and their caregivers. The study comprised two components: a healthcare professional survey and patient and public involvement (PPI) semi-structured discussions. We followed the Strengthening the Reporting of Observational Studies in Epidemiology (STROBE) checklist [20] for the reporting of quantitative data and PPI activities were reported in accordance with the Guidance for Reporting Involvement of Patients and the Public (GRIPP2) short-form reporting checklist [21].

### 2.2. Survey Development and Administration

The questionnaire was adapted from a previously published survey of prehabilitation practices in adult HCT services [22] by a multidisciplinary research team with expertise in paediatric HSCT, nutrition, physical activity, and prehabilitation [22,23]. Content was further informed by a review of the relevant literature to ensure coverage of key domains [6]. To strengthen content validity, the draft instrument was piloted by healthcare professionals with experience in survey design within adult HSCT prehabilitation research [24]. Items were adapted accordingly to enable future comparisons, while ensuring relevance to paediatric practice [22]. As this was a descriptive practice survey rather than a psychometric scale, no formal content-validity index or psychometric validation was undertaken. The survey assessed current pre-transplant practices and perspectives on prehabilitation in paediatric HSCT (Supplementary File S1).

### 2.3. Recruitment, Sampling and Eligibility Criteria

Healthcare professionals working in paediatric cancer care in the UK and Republic of Ireland were recruited through the Children’s Cancer and Leukaemia Group (CCLG), a professional association for paediatric cancer care. The recruitment strategy aimed to obtain responses from healthcare professionals across all 16 paediatric HSCT centres included in the study sampling framework and from a range of professional groups involved in HSCT care. As this was an exploratory descriptive study designed to map current practice rather than testing a prespecified hypothesis, no formal sample-size calculation or numerical recruitment target for individual healthcare professional responses was established.

The survey invitation, participant information sheet and open online survey link were disseminated through the CCLG platform and relevant special interest groups, including the Multiprofessionals Group, Bone Marrow Transplant Physiotherapy and Occupational Therapy Working Group, and National Institute for Health and Care Research Children, Teenagers and Young Adults Group. Because these networks managed their own distribution lists, memberships could overlap, and the invitation could be forwarded within professional networks and participating centres, the number of individual healthcare professionals who received or viewed the invitation could not be determined. Consequently, an individual-level survey response rate could not be calculated. Healthcare professionals were eligible if they provided direct care to CYP requiring HSCT at a paediatric centre in the UK or Republic of Ireland and could respond in English. Those not currently working in paediatric cancer care in these jurisdictions were excluded.

### 2.4. Patient and Public Involvement

Semi-structured discussions with CYP and caregivers experienced in paediatric HSCT complemented the survey and informed intervention development, consistent with stakeholder involvement guidance [25]. Discussions explored preferred timing, setting and delivery modes for prehabilitation, including face-to-face, virtual and individualised approaches.

Participants of varying ages, diagnoses and backgrounds were purposively sampled to capture diverse perspectives [26]. They were recruited from inpatient and outpatient services at one UK paediatric HSCT centre. Discussions occurred during scheduled visits or via secure video call to minimise burden. For participants aged under 16 years, parental consent and developmentally appropriate assent were obtained.

With consent, discussions were audio-recorded, transcribed verbatim and pseudonymised. Recordings were stored on encrypted institutional servers and deleted after transcription.

### 2.5. Ethics and Governance

The survey was distributed through independent professional networks and did not require National Health Service Research Ethics Committee review under Health Research Authority guidance [27], as no NHS patients or patient data were involved. PPI activities followed institutional governance, including voluntary participation, pseudonymisation and the right to withdraw. All researchers held current Disclosure and Barring Service (DBS) checks and Good Clinical Practice (GCP) and safeguarding training and were experienced in working with children and families.

### 2.6. Data Analysis

To address the study aims, quantitative survey data were analysed using descriptive statistics. Categorical and binary variables were summarised as frequencies and proportions, and Likert-scale responses were presented as distributions across response categories. Where appropriate, responses were stratified by professional groups to explore variation in practice and perspectives.

Free-text survey responses were analysed using qualitative content analysis [28]. An inductive approach was used to code responses, and recurring categories were identified to capture common themes relating to current practice, perceived gaps, and implementation challenges [29].

Data from PPI discussions were analysed separately using reflexive thematic analysis. The research team manually conducted thematic analysis to identify recurring themes [30]. Initial inductive coding and theme development were undertaken by H.A. and reviewed by D.R. for coherence and consistency with the transcripts; differences in interpretation were resolved through discussion. Coding, theme development and refinement were documented to support transparency and trustworthiness [31]. Survey and PPI findings were subsequently integrated during interpretation by comparing areas of convergence and complementarity [25]. ChatGPT Pro (OpenAI) supported only theme organisation and wording; no raw or identifiable data were entered, and all coding and interpretation remained with the research team.

## 3. Results

### 3.1. Respondent Characteristics

**Figure 1:**
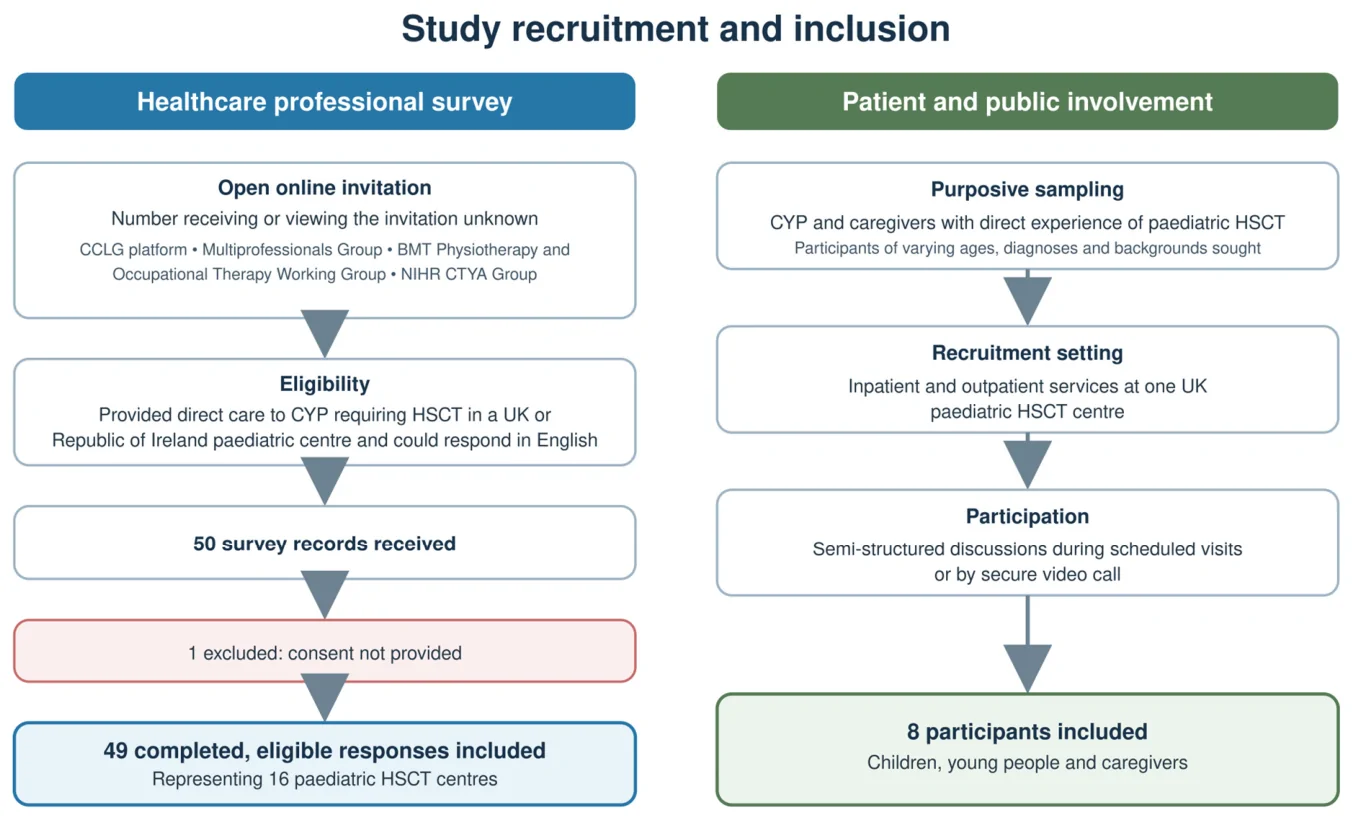
Flow diagram.

A total of 50 survey responses were received (Figure 1). One respondent did not provide consent and did not proceed with the survey, leaving 49 completed and eligible responses for inclusion in the analysis. At least one eligible response was received from each of the 16 paediatric HSCT centres included in the study sampling framework, providing complete centre representation (16/16 centres). The number of respondents contributed by each centre varied.

Respondents comprised a multidisciplinary workforce, including allied health professionals (38.8%), consultants (32.7%), nurses (24.5%), and psychologists (4.1%). Nearly half (49.0%) had more than 10 years of HSCT experience, and most were based in centres delivering both autologous and allogeneic transplants (87.8%). Over three quarters (77.6%) reported working with CYP prior to HSCT. Respondents were geographically distributed across Yorkshire (30.6%), London (14.3%), and Scotland, Northwest England, and Northern England (each 10.2%), with smaller representation from other UK regions and Ireland. Full respondent characteristics are presented in Table 1.

### 3.2. Availability of Prehabilitation Services Across Paediatric HSCT Centres

Of the 49 respondents, 27 (55.1%) reported that a formal prehabilitation service was in place at their centre, whilst 22 (44.9%) reported no such service existed. Responses varied both within and between centres. One centre reported unanimous agreement that no formal service existed, whilst two centres reported full agreement that a service was in place. Within-centre variation in responses regarding service availability was observed across centres.

### 3.3. Pre-HSCT Screening and Assessment

**Figure 2:**
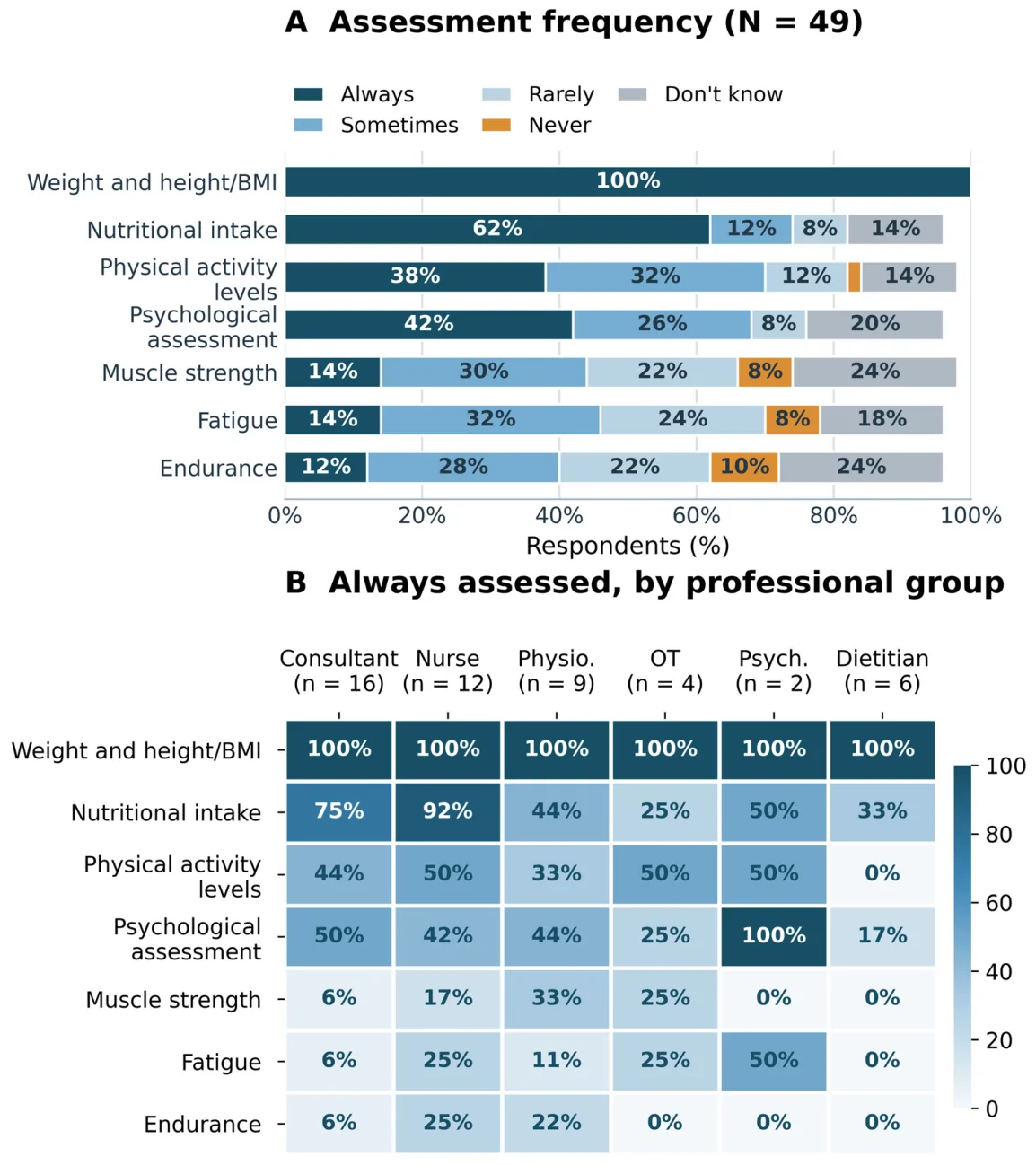
Pre-HSCT screening and assessment practices reported by healthcare professionals (N = 49). (**A**) Frequency of pre-HSCT screening assessments across domains. (**B**) Proportion reporting that each domain was always assessed, stratified by professional group. Percentages may not total 100 because of item non-response and rounding. Abbreviations: BMI, body mass index; HSCT, haematopoietic stem cell transplantation.

Substantial variation in pre-HSCT screening practices was reported across centres (Figure 2A). Weight, height, and BMI were the only domains universally assessed by all respondents, 49 (100%). Nutritional intake was reported as always assessed by 30 (62%) respondents, and psychological assessment by 21 (42%). Physical function domains were less consistently reported. Muscle strength, fatigue, and endurance were reported as always assessed by seven (14%), seven (14%), and six (12%) respondents respectively, with many indicating these were undertaken only sometimes, rarely, or not at all.

Variation in screening practice was also evident across professional groups (Figure 2B). Consultants and nurses reported broader involvement across screening domains, whilst physiotherapists were primarily engaged in physical function assessments and frequently reported uncertainty regarding nutritional and psychological screening. Dietitians reported awareness of nutritional intake assessment in only a third of cases and reported limited awareness of physical function screening, with the majority selecting “don’t know” across muscle strength, fatigue, and endurance domains.

### 3.4. Prehabilitation Advice and Interventions Currently Provided

**Figure 3:**
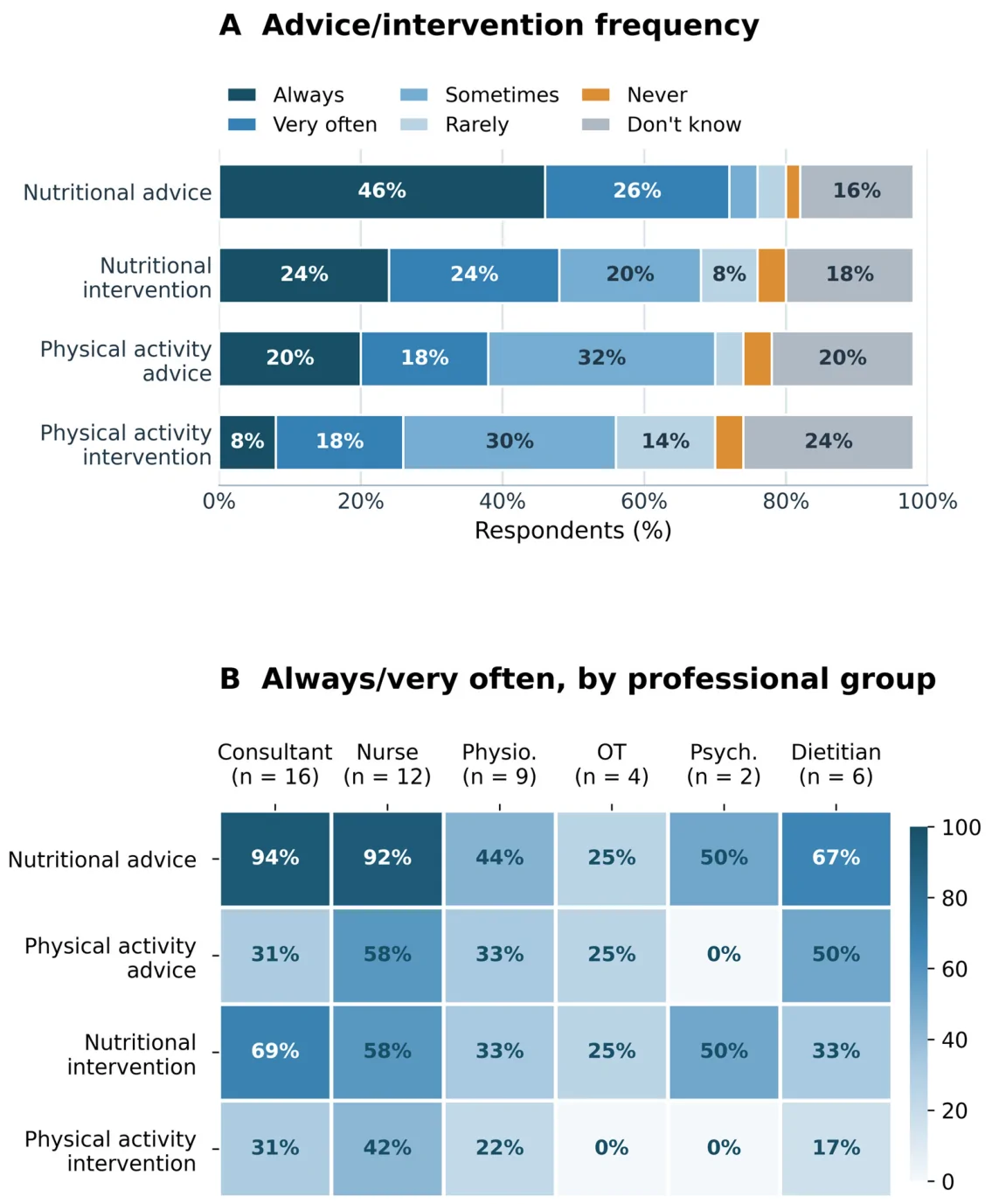
Provision of pre-HSCT advice and interventions reported by healthcare professionals (N = 49). (**A**) Frequency of nutritional and physical activity advice and interventions. (**B**) Proportion reporting that each component was always, or very often provided, stratified by professional group. Percentages may not total 100 because of item non-response and rounding. Abbreviation: HSCT, haematopoietic stem cell transplantation.

Provision of pre-HSCT advice and interventions varied considerably across domains (Figure 3A). Nutritional advice was the most consistently provided, with 36 (72%) respondents reporting always (N = 23, 46%) or very often (N = 13, 26%) delivery. Physical activity advice was less consistently delivered, with 19 (38%) reporting always (N = 10, 20%) or very often (N = 9, 18%) provision, whilst 16 (33%) reported it was only sometimes provided. Structured interventions were less frequently delivered than advice across both domains; nutritional interventions were reported as always or very often delivered by 24 (48%), whilst physical activity interventions were the least consistently provided, with only 13 (26%) reporting always or very often delivery. Notably, 12 (24%) respondents reported not knowing whether physical activity interventions were delivered at their centre.

Provision patterns differed by intervention type and professional group (Figure 3B). Nutritional advice was most consistently reported as always or very often by 15 consultants (94%) and 11 nurses (92%), whilst lower rates were reported by one occupational therapist (25%) and four physiotherapists (44%). Physical activity advice and intervention showed greater variability across all professional groups, with occupational therapists and psychologists reporting 0 (0%) always or very often delivery for physical activity intervention. Half of dietitians (3; 50%) reported providing physical activity advice, while four (67%) provided nutritional advice and two (33%) provided nutritional interventions.

### 3.5. Timing of Introduction of Prehabilitation

**Figure 4:**
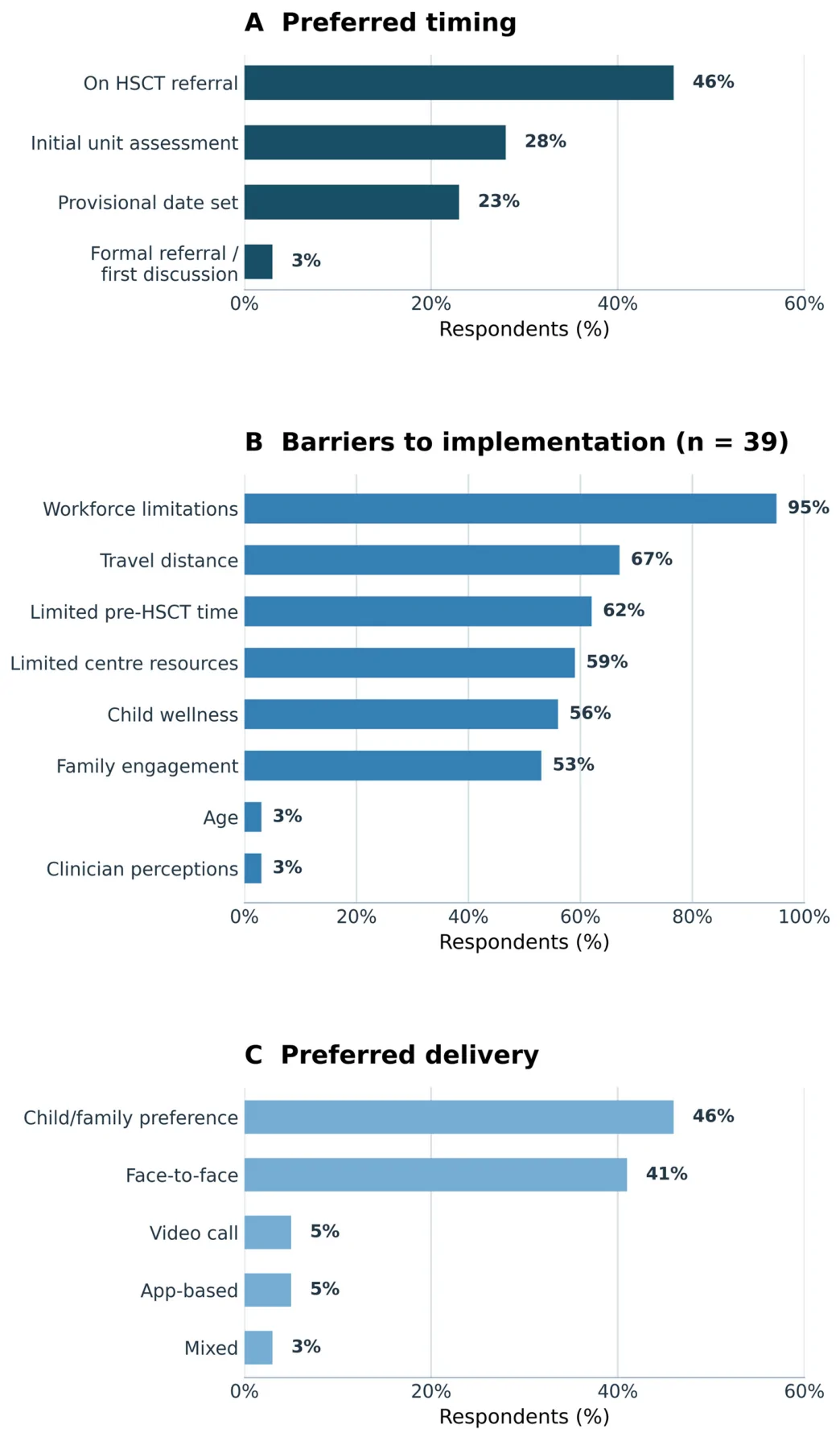
Implementation and delivery of prehabilitation before paediatric HSCT (n = 39). (**A**) Preferred timing of introduction. (**B**) Reported barriers to implementation; multiple responses were permitted. (**C**) Preferred delivery modes. Abbreviation: HSCT, haematopoietic stem cell transplantation.

Of 49 respondents, 39 answered this question (Figure 4A). The majority favoured introduction of prehabilitation at the point of referral for HSCT, 18 (46%), with a further 11 (28%) preferring initial assessment at the HSCT unit and nine (23%) when a provisional transplant date had been set. Only one (3%) respondent suggested introduction at the point of formal referral or first discussion.

### 3.6. Barriers to Implementing Prehabilitation Prior to HSCT

A total of 39 of 49 (79.6%) respondents reported on barriers to implementing prehabilitation prior to HSCT (Figure 4B). Workforce limitations were the most commonly reported barrier, 37 (95%). Travel distance to programmes was identified by 26 (67%) respondents, whilst limited time between treatment decision and initiation of HSCT was reported by 24 (62%). Restricted centre resources were identified by 23 (59%), and concerns regarding the child’s clinical condition or wellness were reported by 22 (56%). Family engagement challenges were reported by 21 (53%). In contrast, age and clinician perceptions were rarely identified as barriers, each reported by only one (3%) respondent.

### 3.7. Delivery Models of Prehabilitation

Of the 49 respondents, 39 (79.6%) answered this question (Figure 4C). Child and family preference was the most frequently endorsed delivery model, 18 (46%), followed by face-to-face delivery, 16 (41%). Remote delivery via video call and app-based platforms were each endorsed by two (5%) respondents, and one (3%) suggested a mixed approach combining multiple delivery methods.

### 3.8. Patient and Public Involvement

A summary of healthcare professional survey and patient and public involvement findings by theme is presented in Table 2.

#### Illustrative Perspectives from PPI Discussions

The following participant accounts illustrate the themes developed through reflexive thematic analysis and the family-centred insights that the HCP survey alone could not capture.

On the gap between intention and access, one participant described wanting to prepare physically for transplant but finding that clinical events moved faster than expected:

*“I was going to ask my dad if we could do some gym stuff, but we were in the hospital quicker—it got in the way.”*

On the importance of person-centred nutritional support, a parent described the contrast between an encounter that felt dismissive and one that felt collaborative:

*“[She] made us feel at ease… she was asking us questions about what he liked and what he didn’t like.”*

On the risk of prescriptive programmes, one participant cautioned that fixed daily requirements could reduce rather than support engagement:

*“If you have a programme you have to do something every day and I feel like that would get a bit exhausting… they’re more opting out of deciding to get up and get around.”*

On the need for honest early preparation, a parent described a distressing situation arising from inadequate procedural information:

*“Tell them what might happen… she got scared the minute she had to have a tube and she refused because she was scared.”*

## 4. Discussion

To our knowledge, this is the first study to examine prehabilitation-related practices across UK and Republic of Ireland paediatric HSCT centres, integrating both healthcare professional and family perspectives. Formal prehabilitation services were reported by just over half of respondents, with substantial within-centre variation suggesting provision is largely informal and profession-led. Nutritional advice was the most consistently delivered component, 36 (72%), whilst physical activity interventions were the least consistently provided, 13 (26%). Assessment of physical function domains was primarily reported by physiotherapists but remained inconsistent even within this group. Workforce limitations were the predominant barrier, identified by 37 (95%) respondents. Despite these gaps, support for routine prehabilitation was unanimous, with the majority preferring introduction at the point of referral. PPI discussions reinforced these findings, with families describing provision as reactive and absent, expressing preference for flexible hybrid delivery, and identifying psychological preparation as essential from the outset.

### 4.1. Prehabilitation Services: Present but Inconsistent

Only over half of respondents (55.1%) reported that a formal prehabilitation service existed at their centre. However, substantial within-centre disagreement was observed, with healthcare professionals from the same institution providing contradictory responses, as similarly reported in adult HCT services [22]. This suggests that prehabilitation may be interpreted or implemented inconsistently(as similarly reported in adult HCT services [22]) with the absence of a shared definition, informal profession-led provision, variable implementation, or limited multidisciplinary awareness and communication [6,22,23]. These findings identified organisational barriers beyond workforce capacity, including unclear ownership, referral processes and pathway integration. Accordingly, the 55.1% represents respondents’ perceptions rather than independently verified centre-level provision.

PPI findings reinforce this, with families describing pre-transplant support as largely reactive and inconsistently available. Several participants reported receiving no formal physiotherapy or dietetic input prior to admission despite being clinically well enough to engage. In contrast, one participant who received structured home-based physiotherapy demonstrated measurable improvements in physical capacity over four weeks, highlighting both the potential of prehabilitation and the inequities in its current delivery. In adults, prehabilitation is better established and typically aligns with the multimodal Macmillan cancer support definition encompassing physical activity, nutrition and psychological support, with evidence of benefit [16]. In paediatric HSCT, however, evidence remains scarce and very few studies implement truly multimodal interventions consistent with this definition [6]. Nonetheless, emerging evidence and strong professional support justify feasibility testing of structured, multimodal prehabilitation in paediatric HSCT [6,22,23].

### 4.2. Pre-HSCT Screening

The screening data presents one of the clearest and most clinically significant findings of this study. Weight and BMI were universally assessed (100%), and nutritional intake was reported as always screened by 62% of respondents, reflecting the established clinical priority placed on anthropometric and nutritional monitoring within intensive treatment pathways. This aligns with existing evidence highlighting that routine nutritional screening is a fundamental component of paediatric oncology care, given the high risk of malnutrition and its association with adverse clinical outcomes [32,33,34,35,36,37,38]. However, these findings should be interpreted with caution as the assessment of weight is likely driven in part by its clinical necessity for chemotherapy dose calculations [39], rather than solely representing a nutritional screening priority. Muscle strength, fatigue and endurance were reported as always assessed by only seven (14%), seven (14%), and six (12%) respondents, highlighting limited routine assessment of physical reserve [6,10,40].

Physical function parameters such as muscle strength, fatigue, and endurance are strongly associated with treatment tolerance, functional decline, and recovery trajectories in paediatric oncology and HSCT populations [6,10,40]. Their inconsistent assessment therefore suggests that current pre-transplant evaluations may overlook early indicators of physiological reserve and vulnerability. The professional group analysis suggests this gap is structurally determined by the absence of a shared multidisciplinary assessment framework. Consultants and nurses reported the broadest involvement across screening domains, whilst physiotherapists were primarily engaged in physical function assessments but frequently reported uncertainty regarding nutritional and psychological screening, indicating limited cross-disciplinary visibility. Most notably, dietitians demonstrated almost no awareness of physical function screening, with the majority selecting “don’t know” for domains including muscle strength, fatigue, and endurance. This is not a reflection of individual practitioners; rather, it suggests that in the absence of a structured multidisciplinary screening protocol [6,10,40] each profession assesses within its recognised remit, resulting in a patchwork of siloed activity that fails to generate a comprehensive picture of pre-transplant vulnerability.

PPI findings reinforce the clinical significance of this gap. Families described uncertainty around safe physical activity levels and, in the absence of guidance, adopted cautious and often inactive approaches. Whilst understandable, this behaviour is likely to exacerbate functional decline, given evidence that physical impairments are already present prior to HSCT and that exercise is safe and beneficial in this population [6,41]. The safety of exercise in paediatric HSCT is, however, an area warranting further formal evaluation, as robust evidence is needed to reassure both families and clinicians and to support integration of physical activity into routine pre-transplant care [6]. Addressing the screening gap is therefore likely to be an important prerequisite for effective prehabilitation; without systematic identification of need, targeted intervention cannot be delivered.

### 4.3. Prehabilitation Provision

Building on the screening gap identified above, findings related to provision further highlight a pronounced imbalance in routine pre-transplant care. Nutritional advice was reported as always or very often provided by 36 (72%) respondents and nutritional interventions by 24 (48%). Malnutrition is a common problem in children with cancer, impacting quality of life and survival [42,43]. Consequently, nutritional support is well established within paediatric oncology care, with clinical guidance recommending routine malnutrition screening and appropriate nutritional intervention where indicated [36,37,38,44], alongside the paediatric oncology literature emphasising the importance of early and ongoing nutritional assessment in CYP with cancer [44,45].

In contrast, physical activity was reported by only 19 (38%) respondents, with a further 16 (33%) indicating it was provided only sometimes. Structured physical activity interventions were delivered by only 13 (26%), and 12 (24%) respondents were uncertain whether such interventions existed within their pathway at all. These findings demonstrate a clear guideline–practice gap: although the 2023 guideline strongly recommends physical activity to manage fatigue and routine fatigue assessment using a validated scale, fatigue was always assessed by only 14% of respondents, while only 38% reported routinely providing physical activity advice and 26% delivered structured physical activity interventions [46]. Whilst physical activity has been shown to improve endurance and strength in children undergoing HSCT, no studies have yet investigated prehabilitation, that is, exercise before HSCT, to improve functional outcomes [6,41,47].

An equally important consideration is the practical feasibility of delivering physical activity prehabilitation in this population. In paediatric HSCT, transplantation is generally reserved for children with high-risk or relapsed disease, typically following prior intensive chemotherapy, meaning that children arrive at transplant with a significant cumulative treatment burden [2,6]. The pre-transplant window may therefore be short, with many CYP still receiving adjunctive treatment and experiencing significant treatment-related illness and fatigue during this period [6]. Furthermore, the paediatric age range of 0 to 18 years encompasses vastly different developmental stages, physical capacities, and family contexts [6,13,47]. Clinical exercise interventions in paediatric oncology are feasible and safe, but the evidence base is heterogeneous and limited largely to specific diagnoses such as acute lymphoblastic leukaemia [48], highlighting the need for individually tailored approaches rather than a single standardised programme. The family context must also be considered, as the demands of treatment affect not only the child but the entire family unit, and any intervention design must accommodate this [6]. Furthermore, PPI findings reinforce this picture. Families described support as reactive and limited, with uncertainty around safe activity levels leading to cautious and often inactive behaviours that may exacerbate existing deconditioning. Where structured, individualised support was available, clear benefits were observed, highlighting both the potential of prehabilitation and the current inequities in its delivery. Addressing this gap will require embedding physical activity into pre-transplant pathways in a manner that is flexible, individually tailored, and responsive to the unique developmental, clinical, and family needs of each child.

### 4.4. Timing, Barriers, and Delivery: Translating Preference into Practice

Most respondents supported early prehabilitation, either at HSCT referral (18; 46%) or initial transplant assessment (11; 28%). Although prehabilitation should begin sufficiently before treatment, short or urgent HSCT pathways may constrain delivery [6,16,23] and intervention periods of 6–12 weeks are recommended, which may be unachievable when transplantation is urgent [49]. Workforce limitations were the dominant barrier (37; 95%), reflecting staff shortages, competing priorities and limited protected time; previous qualitative research similarly identified workforce constraints and the absence of standardised pathways as key implementation barriers [50]. These findings suggest that prehabilitation is not yet embedded within paediatric HSCT pathways and may be deprioritised without clear ownership and integration. Other barriers included travel distance (26; 67%), compressed timelines (24; 62%), limited centre resources (23; 59%), child wellness (22; 56%) and family engagement (21; 53%), whereas age and clinician perceptions were rarely identified (each 1; 3%), indicating predominantly systemic and logistical rather than attitudinal barriers.

Additional implementation challenges include coordinating programme delivery and integrating prehabilitation within existing clinical pathways [50,51]. The distinction is important for informing further intervention design and service development. PPI findings reinforce this picture. Families described support as reactive, inconsistent and dependent on local provision, with limited guidance and difficulty engaging due to treatment burden and logistical challenges. Younger children and their families favoured in-person contact, whilst older children expressed openness to digital delivery, further supporting the need for age-appropriate, flexible delivery models that integrate into rather than add to the demands of the transplant pathway. Collectively, these findings indicate that future paediatric HSCT prehabilitation pathways must prioritise flexibility, accessibility, and integration within routine care to achieve meaningful engagement and sustainable implementation.

### 4.5. Strengths and Limitations

This study has several strengths. The healthcare professional survey included eligible responses from all 16 paediatric HSCT centres within the UK and Republic of Ireland sampling framework, providing complete centre representation and broad geographical coverage. The multidisciplinary respondent profile also enabled exploratory professional group-level analyses that identified patterns that may not have been apparent in a single-profession survey. In addition, integrating PPI alongside the healthcare professional survey provided valuable family-centred perspectives that extended interpretation of the quantitative findings.

However, several limitations must be acknowledged. The survey was disseminated through open professional networks, and the number and characteristics of healthcare professionals who received or viewed the invitation were unknown. An individual-level response rate could therefore not be calculated. Although all 16 centres were represented, the number of respondents per centre varied, and complete centre representation should not be interpreted as demonstrating that the 49 respondents were representative of the wider paediatric HSCT workforce. Self-selection and non-response bias are possible, as healthcare professionals with greater knowledge of or interest in prehabilitation may have been more likely to participate. Consultants and nurses represented most respondents, whereas physiotherapists and dietitians were comparatively underrepresented, potentially limiting insight into physical activity and nutritional practice. Professional group comparisons should therefore be considered exploratory. Furthermore, the cross-sectional survey design cannot confirm that reported practices reflect clinical behaviour, and social desirability bias may have resulted in overestimation of current provision. Furthermore, within-centre discordance across multiple institutions suggests that self-report alone may not reliably indicate formal service availability. Although broadly representative, the sample of 49 respondents was relatively small and limited subgroup analyses. Consultants and nurses represented most respondents, reflecting the composition of many HSCT teams, although physiotherapists and dietitians were comparatively underrepresented, potentially limiting insight into exercise and nutritional practice. Finally, PPI was conducted with a small group from a single centre with limited demographic diversity and sample size does not permit claims of data saturation; therefore, findings may reflect local experiences and may not be transferable to centres with different populations, resources or models of HSCT care.

### 4.6. Implications for Clinical Practice and Future Research Directions

The strong clinical consensus in favour of prehabilitation, alongside the structural gaps identified in this study, suggests that prehabilitation is viewed as an important unmet area of supportive care within paediatric HSCT pathways. However, evidence regarding feasibility, acceptability, and clinical effectiveness in this population remains limited, and further research is required before widespread implementation can be recommended.

Findings from this study provide an important foundation for future intervention development. In particular, the results support the need for standardised multidimensional assessment protocols that extend beyond anthropometric measures to include physical function, fatigue, endurance, and comprehensive nutritional status, enabling consistent pre- and post-transplant evaluation across centres. Importantly, both healthcare professionals and families identified flexibility as central to programme feasibility. PPI findings demonstrated strong interest from CYP and families in supportive prehabilitation approaches, while also highlighting practical considerations regarding timing, communication, treatment burden, and mode of delivery. Younger children and families favoured face-to-face interaction, whereas older children expressed greater openness to digital approaches, supporting further exploration of flexible and hybrid delivery models.

Future research should prioritise centre-level service mapping and qualitative implementation research to examine the reasons for this discordance, and feasibility and pilot interventions are needed to refine programme content, timing, outcome measures, and delivery pathways before progression to adequately powered multicentre randomised controlled trials. National collaboration across paediatric HSCT centres will likely be essential to generate robust evidence regarding effectiveness, implementation, and long-term sustainability.

## 5. Conclusions

This study provides the first systematic examination of prehabilitation practices across paediatric HSCT centres in the UK and Republic of Ireland, revealing that current provision is inconsistent, fragmented, and insufficiently integrated within clinical pathways. Physical function assessment and physical activity provision remain particularly underdeveloped, and provision varies considerably by centre and professional group. Whilst evidence in paediatric HSCT remains limited, clinicians broadly support the principle of prehabilitation, and families identify its absence as a meaningful unmet need. The findings suggest that the challenge is not solely one of awareness but of implementation, ownership, and pathway integration. Future research should prioritise establishing the feasibility and safety of prehabilitation in this population, developing individually tailored and flexibly delivered interventions, and evaluating their effectiveness through well-designed prospective studies. CYP undergoing HSCT deserve the opportunity to be as prepared as possible for one of the most demanding treatments in paediatric medicine.

## Figures and Tables

**Table 1:** Respondent characteristics and those of their associated transplant centres.

Healthcare Professional (HCP) (N = 49)	N (%)
**Profession**	
**Allied Health Professional (N = 19; 38.8%)**	
Physiotherapist	9 (18.4%)
Dietitian	6 (12.2%)
Occupational Therapist	4 (8.2%)
**Consultant (N = 16; 32.7%)**	
HSCT Consultant	10 (20.4%)
Oncologist/Autologous Transplanter	3 (6.1%)
Paediatric Oncology/Haematology Consultant	2 (4.1%)
Associate Specialist	1 (2.0%)
**Nurse (N = 12; 24.5%)**	
HSCT/BMT Specialist Nurse	8 (16.3%)
Advanced Nurse Practitioner	2 (4.1%)
Ward/Inpatient Nurse	2 (4.1%)
**Psychology (N = 2; 4.1%)**	
Psychologist	2 (4.1%)
Proportion working with children prior to HSCT	38 (77.6%)
**Clinical Experience Working with HSCT Patients**	
Greater than 10 years	24 (49.0%)
2–5 years	13 (26.5%)
5–10 years	7 (14.3%)
Less than 2 years	5 (10.2%)
**Type of HSCT Delivered at Centre**	
Both autologous and allogeneic	43 (87.8%)
Autologous only	5 (10.2%)
Allogeneic only	1 (2.0%)
**Transplant Centre (N = 16 centres)**	
**Locality of Respondents’ Centres**	
Yorkshire	15 (30.6%)
London	7 (14.3%)
Scotland	5 (10.2%)
Northwest England	5 (10.2%)
Northern England	5 (10.2%)
Southwest England	3 (6.1%)
Northern Ireland	2 (4.1%)
Wales	2 (4.1%)
Midlands	2 (4.1%)
Republic of Ireland	2 (4.1%)
Eastern England	1 (2.0%)

**Abbreviations:** BMT, bone marrow transplantation; HCP, healthcare professional; HSCT, haematopoietic stem cell transplantation.

**Table 2:** Summary of healthcare professional survey and patient and public involvement findings by theme.

Theme	HCP Survey Findings	PPI Findings
**Current Provision**	27 (55.1%) reported a formal prehabilitation service existed, with substantial within-centre variation suggesting provision is largely informal and profession-led	Families described pre-transplant support as largely absent or reactive; several reported never seeing a dietitian before transplant, with physiotherapy input inconsistent across referring centres
**Nutritional Support**	Nutritional advice was the most consistently delivered component, with 36 (72%) reporting always or very often delivery; dietitians reported limited awareness of physical function screening	Dietitian involvement was largely reactive, triggered by weight loss or requirement for tube feeding rather than proactive assessment; families described receiving generic advice rather than individualised guidance; the manner of delivery significantly influenced family engagement
**Physical Activity Support**	Muscle strength, fatigue, and endurance were reported as always assessed by only 7 (14%), 7 (14%), and 6 (12%) respondents respectively; physical activity interventions were the least consistently provided, 13 (26%)	Families valued physical activity support highly; one child progressed from 3 to 10 min on a static bike over four weeks of home visits; several families reported wishing more had been done earlier
**Psychological Preparation and Information Sharing**	Psychological assessment was reported as always undertaken by 21 (42%) respondents, remaining variable across centres and professional groups	Families emphasised the importance of honest, early preparation for procedures and potential complications; one parent described inadequate preparation leading to a child refusing a nasogastric tube out of fear; uncertainty about what to expect was identified as a significant source of distress
**Timing of Prehabilitation**	18 (46%) favoured introduction of a programme at the point of referral for HSCT; 11 (28%) preferred initial assessment at the HSCT unit	Families described the window between diagnosis and transplant admission as shorter than expected; one participant noted “we were in the hospital quicker—it got in the way” when describing plans to prepare physically before admission
**Delivery Model**	16 (41%) endorsed face-to-face delivery; only 2 (5%) endorsed remote delivery, suggesting under-representation of appetite for digital tools	Preferences varied by age; younger children and families favoured in-person contact, whilst older children expressed openness to app-based or video call delivery; gamification, reward systems, and activity menus were consistently highlighted as important features to support engagement
**Flexibility and Child-Led Approach**	No standardised model of pre-transplant optimisation currently exists; within-centre variation suggests ad hoc delivery	Children consistently described fixed daily programmes as unsustainable; a menu-based approach allowing activity choice based on daily capacity was preferred; one participant cautioned “if you have a programme you have to do something every day, I feel like that would get a bit exhausting”
**Family and Sibling Inclusion**	Family engagement challenges were identified as a barrier by 21 (53%) respondents	Families highlighted the importance of including siblings and incorporating prehabilitation into family activities rather than creating additional appointments; one single parent described precious family time at home as a priority that any intervention would need to accommodate

**Abbreviations:** HCP, healthcare professional; HSCT, haematopoietic stem cell transplantation; PPI, patient and public involvement.

## Data Availability

The data presented in this study are available on request from the corresponding author due to ethical restrictions relating to participant confidentiality and the anonymity of PPI participants.

## Supplementary Materials

The following supporting information can be downloaded at: https://www.mdpi.com/article/10.3390/nu18172762/s1, Supplementary File S1: Healthcare professional questionnaire.

## Abbreviations

BMI, body mass index; BMT, bone marrow transplantation; CCLG, Children’s Cancer and Leukaemia Group; CTYA, Children, Teenagers and Young Adults; CYP, children and young people; DBS, Disclosure and Barring Service; GCP, Good Clinical Practice; GRIPP2, Guidance for Reporting Involvement of Patients and the Public; GvHD, graft-versus-host disease; HCP, healthcare professional; HLA, human leukocyte antigen; HSCT, haematopoietic stem cell transplantation; MDT, multidisciplinary team; NIHR, National Institute for Health and Care Research; PPI, patient and public involvement.
