# Supplementary material for: Prehabilitation Practices for Paediatric Haematopoietic Stem Cell Transplantation: A Survey and Patient and Public Involvement Study of Healthcare Professionals
Source: Nutrients. 2026 Aug 24;18(17):2762. doi: 10.3390/nu18172762 (PMC13567236; doi:10.3390/nu18172762)
Supplement: Supplementary file 1 [file nutrients-18-02762-s001.zip › nutrients-4473546-supplementary.pdf]

SUPPLEMENTARY FILE S1

Prehabilitation for Children Requiring Haematopoietic Stem Cell Transplantation Following a Diagnosis of Cancer

Healthcare Professional Questionnaire

Completion guidance

Please answer with reference to your current paediatric HSCT service. Select one response unless the question states 'select all that apply'. *No participant-response data are included in this supplementary file.*

Abbreviations: BMI, body mass index; CYP, children and young people; HSCT, haematopoietic stem cell transplantation.

A. Consent

1. I consent to my involvement in this survey. *Select one response.*

- ☐ Yes
- ☐ No

If No, please do not continue with the questionnaire.

B. Centre and Professional Background

2. Does the centre in which you currently work provide a prehabilitation service for children requiring HSCT? *Select one response.*

- ☐ Yes
- ☐ No

3. At which paediatric transplant centre do you work?

Centre: ______________________________

4. What is your occupation or professional role?

Role: ______________________________

5. What type(s) of HSCT does your centre deliver? *Select one response.*

- ☐ Allogeneic HSCT
- ☐ Autologous HSCT
- ☐ Both allogeneic and autologous HSCT

6. How much experience do you have working with children who require HSCT? *Select one response.*

- ☐ Less than 2 years
- ☐ 2–5 years
- ☐ 5–10 years
- ☐ More than 10 years

7. At which stages do you work with children? *Select all that apply.*

- ☐ Before they require HSCT as part of their treatment
- ☐ From referral into the HSCT pathway
- ☐ During their inpatient admission for HSCT
- ☐ During post-HSCT follow-up

☐ When they transition into late-effects services

C. Current Pre-HSCT Screening and Assessment

8. Before admission for HSCT, how often do children and young people receive each of the following screening assessments? *Select one response in each row.*

Screening assessment	Always	Sometimes	Rarely	Never	I don't know
Weight and height/BMI	☐	☐	☐	☐	☐
Nutritional intake	☐	☐	☐	☐	☐
Physical activity levels	☐	☐	☐	☐	☐
Muscle strength	☐	☐	☐	☐	☐
Fatigue	☐	☐	☐	☐	☐
Endurance	☐	☐	☐	☐	☐
Psychological assessment	☐	☐	☐	☐	☐

9. Please list any outcome measures used in these assessments, including questionnaires, scales and measurements.

Response

10. Which professional groups are involved in pre-HSCT assessment? *Select all that apply.*

☐ Consultants
☐ Nursing staff
☐ Physiotherapists
☐ Occupational therapists
☐ Dietitians
☐ Psychologists
☐ Other (please specify): ___________________________________

D. Current Advice and Intervention Before HSCT

11. Before HSCT, how often are children and young people provided with each of the following? *Select one response in each row.*

Advice or intervention	Always	Very often	Sometimes	Rarely	Never	I don't know
Nutritional advice	☐	☐	☐	☐	☐	☐
Physical activity/exercise advice	☐	☐	☐	☐	☐	☐
Nutritional intervention	☐	☐	☐	☐	☐	☐
Physical activity/exercise intervention	☐	☐	☐	☐	☐	☐

E. Views on Routine Prehabilitation

12. Do you think prehabilitation should be considered as part of routine HSCT pathways in the future? *Select one response.*

- ☐ Yes
- ☐ Only for children with an identified need following screening

13. What characteristics would identify a need for further intervention?

Response

14. Should physical activity/exercise advice be provided routinely to CYP before HSCT? *Select one response.*

- ☐ Yes
- ☐ No
- ☐ I don't know

15. Should a physical activity/exercise programme be provided routinely before HSCT? *Select one response.*

- ☐ Yes
- ☐ Only when a problem or need is identified
- ☐ No
- ☐ I don't know

16. Should nutrition be assessed routinely before HSCT? *Select one response.*

- ☐ Yes
- ☐ No
- ☐ I don't know

17. Should nutritional advice be provided routinely before HSCT? *Select one response.*

- ☐ Yes
- ☐ No
- ☐ I don't know

18. Should a routine supportive feeding programme be provided before HSCT? *Select one response.*

- ☐ Yes
- ☐ Only for children at risk of undernutrition
- ☐ No
- ☐ I don't know

F. Timing, Implementation and Delivery

19. When in the HSCT pathway could a prehabilitation intervention be introduced? *Select one response.*

- ☐ When HSCT is first discussed or considered likely by the clinical team
- ☐ On formal referral for HSCT
- ☐ At the initial assessment at the HSCT unit

☐ When a provisional date for HSCT is available

20. What would be the barriers to implementing a prehabilitation intervention? *Select all that apply.*

- ☐ Workforce limitations, including professional time
- ☐ Limited time between the decision to treat and commencement of treatment
- ☐ Distance from the treatment centre limiting child/family access
- ☐ Child/family engagement
- ☐ Child wellness
- ☐ Limited resources at the treatment centre (e.g., space or funding)
- ☐ Other (please specify): ___________________________________

21. Are there any facilitators that should be considered when developing a prehabilitation intervention?

Response

22. What interventions should be included in a prehabilitation programme? *Select all that apply.*

- ☐ Nutritional advice
- ☐ Nutritional intervention
- ☐ Physical activity/exercise advice
- ☐ Health-behaviour advice (e.g., smoking cessation)
- ☐ Psychosocial support
- ☐ Other (please specify): ___________________________________

23. How could prehabilitation interventions best be delivered through your centre? *Select one response.*

- ☐ According to child/family preference
- ☐ Face-to-face in the community
- ☐ Face-to-face in the hospital
- ☐ Multiple methods (e.g., an initial face-to-face appointment followed by videos and/or an app)
- ☐ Via an app
- ☐ Via video call

24. Further comments

Response

End of questionnaire

STROBE Statement—Completed Checklist for a Cross-Sectional Study

Manuscript assessed: Prehabilitation Practices for Paediatric Haematopoietic Stem Cell Transplantation: A Survey and Patient and Public Involvement Study of Healthcare Professionals
Reference version: 27-page combined single-file Nutrients submission: article text and back matter on pp. 1–17, Supplementary File S1 questionnaire on pp. 18–21, references on pp. 22–24, and this STROBE checklist on pp. 25–27. Figures 1–3 are embedded from verified 600-dpi files. Unreported/N/A items are flagged without inventing details. Asterisks denote items for which STROBE advises separate reporting for exposed/unexposed groups; this is not applicable to the descriptive survey.

Section / topic	Item No	Recommendation	Reported on page(s) / location
Title and abstract	1	(a) Indicate the study's design with a commonly used term in the title or the abstract	p. 1 (title and structured Abstract—Methods: cross-sectional survey).
		(b) Provide in the abstract an informative and balanced summary of what was done and what was found	p. 1 (structured Abstract: Background and Objectives, Methods, Results and Conclusions).
**Introduction**			
Background/rationale	2	Explain the scientific background and rationale for the investigation being reported	pp. 2–3 (Introduction).
Objectives	3	State specific objectives, including any prespecified hypotheses	p. 3 (final paragraph of Introduction); also p. 1 (Abstract—Background and Objectives).
**Methods**			
Study design	4	Present key elements of study design early in the paper	p. 3 (§2.1 Study Design).
Setting	5	Describe the setting, locations, and relevant dates, including periods of recruitment, exposure, follow-up, and data collection	pp. 3–4 (§§2.1–2.4: UK and Republic of Ireland; survey February–April 2025; complementary PPI at one UK paediatric HSCT centre, in person or by secure video call).
Participants	6	(a) Give the eligibility criteria, and the sources and methods of selection of participants	p. 3 (§2.3: professional-network recruitment and survey eligibility/exclusion criteria); pp. 3–4 (§2.4: complementary PPI sampling and recruitment).
Variables	7	Clearly define all outcomes, exposures, predictors, potential confounders, and effect modifiers. Give diagnostic criteria, if applicable	p. 3 (§2.2: survey domains, operational definitions of advice/interventions and response formats); Supplementary File S1 (full questionnaire).
Data sources/ measurement	8*	For each variable of interest, give sources of data and details of methods of assessment (measurement). Describe comparability of assessment methods if there is more than one group	p. 3 (§2.2: Qualtrics survey, response formats and operational definitions); Supplementary File S1. PPI data collection is described on pp. 3–4 (§2.4).
Bias	9	Describe any efforts to address potential sources of bias	pp. 3 and 15–16 (multidisciplinary development/piloting and broad professional-network recruitment; remaining self-report, social-desirability, representation and single-centre PPI limitations are acknowledged).
Study size	10	Explain how the study size was arrived at	Not explicitly reported: no a priori sample-size calculation is provided. Recruitment approach/period is on p. 3; the achieved eligible sample (n = 49) is on p. 4.
Quantitative variables	11	Explain how quantitative variables were handled in the analyses. If applicable, describe which groupings were chosen and why	pp. 3–4 (§§2.2 and 2.6: categorical, binary and Likert variables summarised as frequencies/proportions and response distributions); category boundaries are also shown in Supplementary File S1 and Table 1 (pp. 4–5).
Statistical methods	12	(a) Describe all statistical methods, including those used to control for confounding	p. 4 (§2.6: descriptive statistics and qualitative content analysis; PPI analysed separately using reflexive thematic analysis).

Section / topic	Item No	Recommendation	Reported on page(s) / location
		(b) Describe any methods used to examine subgroups and interactions	p. 4 (§2.6: stratification by professional group); subgroup results pp. 5–8 and Figures 1B/2B (pp. 7 and 8).
		(c) Explain how missing data were addressed	Not explicitly described in Methods. Item-specific denominators/non-response are reported in Results (pp. 9–10: n = 39/49) and the Figure 3 legend (p. 10).
		(d) If applicable, describe analytical methods taking account of sampling strategy	Not applicable—no complex sampling design, weighting or cluster-adjusted analysis. Recruitment is described on p. 3.
		(e) Describe any sensitivity analyses	Not applicable—no sensitivity analyses were conducted or reported.

Section / topic	Item No	Recommendation	Reported on page(s) / location
**Results**			
**Participants**	13*	(a) Report numbers of individuals at each stage of study—eg numbers potentially eligible, examined for eligibility, confirmed eligible, included in the study, completing follow-up, and analysed	p. 4 (§3.1: 50 responses received, one excluded for no consent, 49 eligible/analysed); Table 1, pp. 4–5.
		(b) Give reasons for non-participation at each stage	p. 4 (§3.1: one respondent excluded because consent was not provided).
		(c) Consider use of a flow diagram	Not used; participant accounting is reported in text on p. 4. The cross-sectional survey had no follow-up stages.
**Descriptive data**	14*	(a) Give characteristics of study participants (eg demographic, clinical, social) and information on exposures and potential confounders	pp. 4–5 (§3.1 and Table 1: profession, experience, transplant type and centre locality).
		(b) Indicate number of participants with missing data for each variable of interest	Item-specific denominators are reported where relevant (pp. 9–10: 39 of 49 for timing, barriers and delivery; Figure 3 legend, p. 10). A variable-by-variable missing-data table is not provided.
**Outcome data**	15*	Report numbers of outcome events or summary measures	pp. 5–12 (§§3.2–3.8); Figures 1–3, pp. 7, 8 and 10; Tables 1–2, pp. 4–5 and 12.
**Main results**	16	(a) Give unadjusted estimates and, if applicable, confounder-adjusted estimates and their precision (eg, 95% confidence interval). Make clear which confounders were adjusted for and why they were included	pp. 5–12 (unadjusted frequencies/proportions); Figures 1–3, pp. 7, 8 and 10; Tables 1–2, pp. 4–5 and 12. Adjusted estimates/confounder control were not applicable.
		(b) Report category boundaries when continuous variables were categorized	Response categories are defined on p. 3 and in Supplementary File S1; experience and transplant-type categories appear in Table 1, pp. 4–5.
		(c) If relevant, consider translating estimates of relative risk into absolute risk for a meaningful time period	Not applicable—no relative-risk estimates were calculated.
**Other analyses**	17	Report other analyses done—eg analyses of subgroups and interactions, and sensitivity analyses	Professional-group subgroup analyses: pp. 5–8 and Figures 1B/2B (pp. 7 and 8). No interaction or sensitivity analyses were reported.
**Discussion**			
**Key results**	18	Summarise key results with reference to study objectives	pp. 12–13 (opening of Discussion), p. 16 (Conclusions), and Table 2, p. 12.
**Limitations**	19	Discuss limitations of the study, taking into account sources of potential bias or imprecision. Discuss both direction and magnitude of any potential bias	pp. 15–16 (§4.5 Strengths and Limitations: self-report/social-desirability bias, small sample, professional imbalance, single-centre PPI and limited demographic diversity; likely overestimation is noted).
**Interpretation**	20	Give a cautious overall interpretation of results considering objectives, limitations, multiplicity of analyses, results from similar studies, and other relevant evidence	pp. 12–16 (§4 Discussion and §5 Conclusions), interpreted in relation to the objectives, limitations and relevant evidence.
**Generalisability**	21	Discuss the generalisability (external validity) of the study results	pp. 15–16 (§4.5: 16-centre/multidisciplinary coverage and limits to representativeness); p. 16 (§4.6: implications for national multicentre research).
**Other information**			
**Funding**	22	Give the source of funding and the role of the funders for the present study and, if applicable, for the original study on which the present article is based	p. 17 (Funding: CCLG support, researcher time/resources and participant tokens). No further funder role in design, analysis or reporting is explicitly stated.
